# Treatment of dysferlinopathy with deflazacort: a double-blind, placebo-controlled clinical trial

**DOI:** 10.1186/1750-1172-8-26

**Published:** 2013-02-14

**Authors:** Maggie C Walter, Peter Reilich, Simone Thiele, Joachim Schessl, Herbert Schreiber, Karlheinz Reiners, Wolfram Kress, Clemens Müller-Reible, Matthias Vorgerd, Peter Urban, Bertold Schrank, Marcus Deschauer, Beate Schlotter-Weigel, Ralf Kohnen, Hanns Lochmüller

**Affiliations:** 1 Friedrich-Baur-Institute, Department of Neurology, Ludwig-Maximilians-University of Munich, Ziemssenstr. 1a, Munich, 80336, Germany; 2 Neuropoint Patient Academy, Ulm, Germany; 3 Department of Neurology, University of Würzburg, Würzburg, Germany; 4 Department of Human Genetics, Biozentrum, University of Würzburg, Würzburg, Germany; 5 Department of Neurology, Neuromuscular Center Ruhrgebiet, Ruhr-University Bochum, Bochum, Germany; 6 Department of Neurology, Asklepios Klinik Barmbek, Hamburg, Germany; 7 Department of Neurology, Deutsche Klinik für Diagnostik, Wiesbaden, Germany; 8 Department of Neurology, University Halle-Wittenberg, Halle, Germany; 9 RPS Research Germany GmbH, Nürnberg, Germany; 10 Institute of Genetic Medicine, Newcastle University, Newcastle-upon-Tyne, UK

**Keywords:** Limb girdle muscular dystrophy (LGMD), Dysferlinopathy, Therapy, Deflazacort, Muscle strength, Steroids

## Abstract

**Background:**

Dysferlinopathies are autosomal recessive disorders caused by mutations in the dysferlin (*DYSF*) gene encoding the dysferlin protein. DYSF mutations lead to a wide range of muscular phenotypes, with the most prominent being Miyoshi myopathy (MM) and limb girdle muscular dystrophy type 2B (LGMD2B).

**Methods:**

We assessed the one-year-natural course of dysferlinopathy, and the safety and efficacy of deflazacort treatment in a double-blind, placebo-controlled cross-over trial. After one year of natural course without intervention, 25 patients with genetically defined dysferlinopathy were randomized to receive deflazacort and placebo for six months each (1 mg/kg/day in month one, 1 mg/kg every 2nd day during months two to six) in one of two treatment sequences.

**Results:**

During one year of natural course, muscle strength declined about 2% as measured by CIDD (Clinical Investigation of Duchenne Dystrophy) score, and 76 Newton as measured by hand-held dynamometry. Deflazacort did not improve muscle strength. In contrast, there is a trend of worsening muscle strength under deflazacort treatment, which recovers after discontinuation of the study drug. During deflazacort treatment, patients showed a broad spectrum of steroid side effects.

**Conclusion:**

Deflazacort is not an effective therapy for dysferlinopathies, and off-label use is not warranted. This is an important finding, since steroid treatment should not be administered in patients with dysferlinopathy, who may be often misdiagnosed as polymyositis.

**Trial registration:**

This clinical trial was registered at http://www.ClincalTrials.gov, identifier: NCT00527228, and was always freely accessible to the public.

## Introduction

Dysferlinopathies are autosomal recessive muscular dystrophies caused by mutations in the gene encoding dysferlin (*DYSF*; Online Mendelian Inheritance in Man [OMIM] gene number 603009, Chr 2p13, GenBank NM_003494.2) [1,2]. Dysferlinopathies are rare muscular dystrophies, as the number of adult patients is estimated between 1/100,000 to 1/200,000 [3]. Miyoshi myopathy [4] (MM) and distal anterior compartment myopathy [5] (DMAT), both allelic distal muscle disorders that preferentially affect the gastrocnemius or tibial muscle, and limb girdle muscular dystrophy (LGMD) type 2B with characteristic proximal weakness at onset [1,2] represent different phenotypic presentations of dysferlinopathy. Dysferlinopathy arises from mutations in the dysferlin gene, which contains 55 exons and spans a region of 150 kb. The clinical picture is usually less severe than most other autosomal recessive LGMD forms, although there is a wide intra- and interfamilial variability [6]. Confinement to wheelchair may occur, on average 10–20 years after onset of the disease. Cardiac and respiratory muscles are not involved in the majority of patients, and patients have normal intelligence. Serum creatine kinase (CK) is always considerably elevated even in the pre-clinical stages. Muscle biopsies typically show dystrophic patterns, additionally, inflammatory changes are frequently seen [7].

Protein analyses in LGMD2B usually show a total deficiency of dysferlin, both through immunohistochemistry and immunoblotting, but partial deficiency is observed in some patients. A large mutational spectrum with more than 400 different sequence variants in the *DYSF* gene has been reported [8]. However, identical mutations in patients even within the same family expressing LGMD2B or MM phenotypes may suggest a role for modifier genes [9].

So far, the natural course of the disease and the mean decline in muscle strength and daily-life activities have only been described in a larger cohort of clinically, but not genetically identified dysferlinopathy patients [10].

Steroids have proven efficacy in Duchenne muscular dystrophy (DMD) [11-13], possibly due, at least in part, to the anti-inflammatory effect of the drug. However, immunosuppressive agents such as azathioprine and cyclosporine did not result in a similar increase of muscle strength as observed with steroids [14,15]. It was hypothesized that steroids may have other or additional modes of action in DMD such as a membrane-stabilizing effect [16]. Dysferlinopathies show marked inflammation in muscle biopsy [7]. Anecdotal reports have suggested that steroids do not improve muscle strength in dysferlinopathy patients, but this was not fully explored in a randomized, controlled trial [17].

This clinical trial was conducted for assessment of the natural course of the disease during a one-year pre-treatment phase and for evaluation of efficacy and side effects of deflazacort in patients with dysferlinopathy. We wanted to assess if deflazacort treatment results in improvement of muscle strength and function compared to placebo.

## Methods

### Study population and inclusion criteria

**Figure 1:**
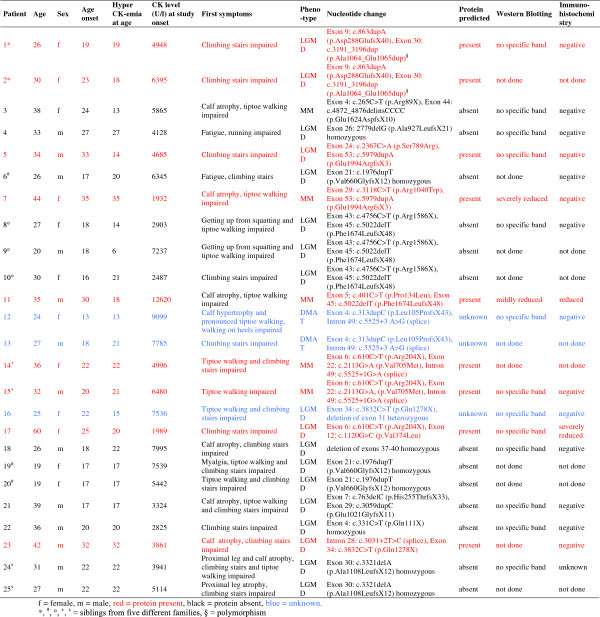
Description of the study population.

25 patients (12 male, 13 female) from 18 different families between 20 and 60 years (mean age 32.4 ± 9.2) with clinical, immunohistochemical, immunoblot and genetic criteria of dysferlinopathy were included in the clinical trial. Three patients presented with MM, two with DMAT phenotype, while the remaining 20 patients mainly showed LGMD involvement. Eight patients had homozygous, 15 patients compound heterozygous mutations in the dysferlin gene; in two patients (patient 1 and 2, siblings), only one heterozygous mutation was detected, since the second described genetic modification may only represent a polymorphism. Within the patients with compound heterozygous mutations, deletion of exon 31 was detected in one patient. In two patients, three compound heterozygous pathogenic dysferlin mutations were detected (see patient details in Figure 1). HyperCKemia was noted in nine of our patients between 4 and 19 years prior to disease onset. Patients <18 years of age, confined to bed or wheelchair, with other neurologic or internal diseases or with former or current steroid treatment were not included. Criteria for premature discontinuation of the trial were withdrawal of informed consent or lack of compliance.

The clinical trial was approved by regulatory authorities and ethics committees at the Ludwig-Maximilians University of Munich and conducted in accordance with the Good Clinical Practice Guidelines. This clinical trial was registered at http://www.ClincalTrials.gov, identifier: NCT00527228 and was always freely accessible to the public. The objectives, study design, risks and benefits of participation were explained to all patients and parents, and their written informed consent was obtained before enrolment.

### Study-design

The clinical trial is designed as a prospective, double-blind, randomized, placebo-controlled cross-over trial. The cross-over design was chosen for several reasons: a) patients with dysferlinopathy are extremely rare, therefore, a sufficient power can be reached with a small sample size by the chosen design, b) in a cross-over design, each patient can serve as his/her own control, c) the willingness to take part in a clinical study is much higher in a cross-over design, since each patient will receive the study drug. In a 12-months pre-treatment phase, the natural history of the disease was assessed. The 12-month visit served as a baseline control prior to treatment; during the two cross-over treatment periods response to treatment was evaluated by comparing evaluations at start and end of each period. All examinations took place at the principal investigator’s center, the other six participating centers helped with patient recruitment and transfer to the clinical trial center.

**Figure 2:**
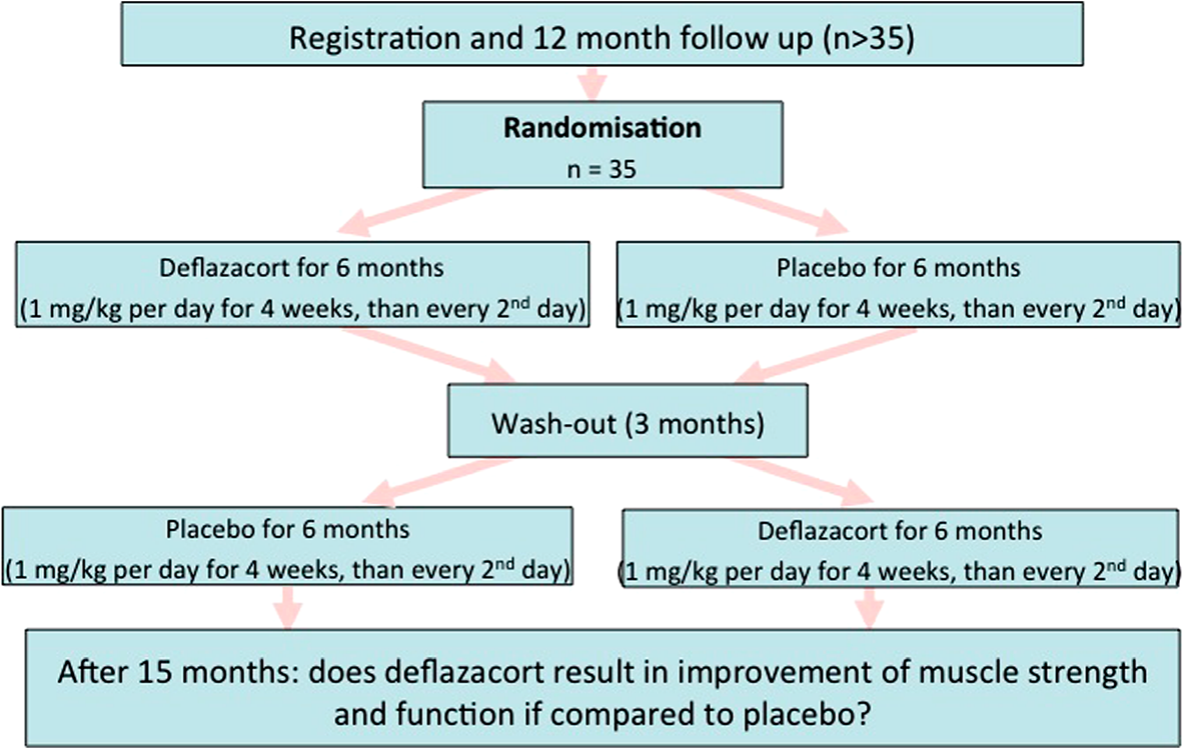
Flow chart of the clinical trial.

All patients had a one-year follow-up of natural history with examinations in six-months intervals prior to the treatment phase. After one year, patients were randomized to six months deflazacort or placebo; after a three-months wash-out, there was cross-over to the alternate treatment (Figure 2).

### Study drug

Since weight gain could be less pronounced in deflazacort treatment compared to other steroids [13], we decided for deflazacort as study drug, and for an alternate day regimen (month 2 to month 6: 1 mg/kg every 2nd day) after the loading phase (month 1: 1 mg/kg/day), since significant improvement of strength along with only mild side effects have been reported with this treatment schedule [11,18]. In contrast to the results of alternate-day therapy with prednisone (1.25 and 2.5 mg/kg every other day) [19], Angelini et al. [18] used a dosage of deflazacort of 2.0 mg/kg every other day and showed sustained improvement over 2 years, including improvement in average muscle strength and in timed function testing [20].

## Methods

Testing was performed by two experienced neurologists after sufficient training with clinical trial procedures. The investigators in the clinical trial center were trained by the MD-NET Clinical Trials Coordination Centre (CTCC) (http://www.md-net.org), inter-rater and intra-rater variability was assessed prior to the clinical trial, and reassessed 12-monthly during the trial period on the enrolled dysferlinopathy patients; the results did now show a relevant variability between tests.

During the pre-treatment phase in 6-month-intervals the natural history of the disease was assessed, and before and after each treatment period the response to treatment was evaluated, using primary and secondary outcome measures.

Primary outcome measures were *manual muscle strength assessed bilaterally by the modified Medical Research Council Scales (MRC) CIDD (Clinical Investigation of Duchenne Dystrophy) score*[21], graded from 0 (worst) to 10 (best) (http://www.researchrom.com/masterlist/view/4), and *quantitative strength assessed by hand-held dynamometry*[22]*(HHD)* (Citec, Groningen, NL) in the same muscle groups (deltoid, biceps brachii, brachioradialis, triceps brachii, hand extensors and flexors, finger extensors and flexors, neck extensors and flexors, quadriceps femoris, iliopsoas, glutaeus maximus, hamstring muscles, tibial anterior muscles, gastrocnemius). HHD was measured in N (Newton), results were indicated as maximum N. Measurements were repeated three times, and the best result was valued.

Secondary outcome measures were *quantitative muscle strength measurement assessed by torque measurement*[23]*(QSMT)*, (M3diagnos, Schnell Company, Germany) (http://www.researchrom.com/masterlist/view/17), *Neuromuscular Symptoms Score*[24]*(NSS)* (http://www.researchrom.com/masterlist/view/9), *Timed Function Tests*[25], *Vignos Scale*[26], *Hammersmith Motor Ability Score*[27], *Clinical Global Impressions (CGI)*[28] and *Quality of Life by SF-36 German version*[29] (http://www.researchrom.com/masterlist/view/54).

*Quantitative Strength Measurement Testing (QSMT)* was assessed using the Multi Muscle Tester M3 Diagnos System. Maximum bilateral strength of biceps and quadriceps muscle was measured isometrically by torque measurement; results were indicated as% of maximum torque difference. Measurements were repeated three times, the best result was valued.

*Handgrip strength* was measured using the Jamar Hydraulik  Handgrip (Sammons Priston Company, Bolingbrook, Ireland). Grip strength can be assessed from 0 to 90 kg, and the maximum value is recorded automatically.

Daily-life activities were evaluated by the *Neuromuscular Symptom and Disability Functional Score (NSS)*. The NSS lists 14 daily-life activities and grades them from no disability to severe disability in 4 categories (total score: 0 = severe disability, 42 = no disability).

*Timed tests* comprised of getting up from lying and sitting position, running 10 meters and climbing four stairs and were measured by stop watch.

The *Vignos scale* grades the patient’s walking ability from 1 to 9 (1 = independent walking and climbing stairs, 9 = wheelchair-bound).

### Hammersmith motor ability score (HMAS)

The scale consists of 20 items (patient lifts head, rolls prone to supine / supine to prone, gets to sitting from lying, sits, gets up from floor, stands, stands on heels/toes/one leg, hops on one leg, gets up from a chair, walks upstairs/downstairs) each scored on a 3 point scoring system. Each activity scores 2 for unaided, 1 for assistance and 0 for inability. The only exceptions are the activities of lying from sitting and lifting the head from prone, in which 1 cannot be scored. A total score can be achieved by summing the scores for all the individual items. The total score can range from 0, if all activities fail, to 40, if all the activities are achieved.

The *Clinical Global Impression (CGI)* is rated by the investigator on a 4-7-point scale, with the severity of illness scale using a range of responses from 1 (normal) through to 7 (amongst the most severely ill patients). CGI change scores range from 1 (very much improved) through to 7 (very much worse).

The health-related *SF-36* is a multi-purpose, short-form health survey with 36 questions. It yields an 8-scale profile of functional health and well-being scores as well as psychometrically-based physical and mental health summary measures. It is a generic measure, as opposed to one that targets a specific age, disease, or treatment group. Accordingly, the SF-36 has proven useful in surveys of general and specific populations, comparing the relative burden of diseases, and in differentiating the health benefits produced by a wide range of different treatments (http://www.sf-36.org).

### Laboratory testing

On each study visit, safety laboratory testing for selected electrolytes, liver, kidney and inflammation analytics and hematology variables (sodium, potassium, creatinine, urea, ASAT, ALAT, GGT, CK, ESR, CRP, blood cell count) were performed, and adverse events were recorded. Genetic testing for dysferlin was carried out for all patients by the Department of Human Genetics at the University of Würzburg. All 55 coding exons and flanking splice regions of the dysferlin gene were amplified with exon-specific primers, followed by direct sequencing on both strands. If only one mutation was found by sequencing, gene dosis was measured for most of the exons using the MLPA kit P268-A1 from MRC Holland.

### Statistics

Randomization to the two sequences of treatment was done by the MD-NET Clinical Trials Coordination Centre (CTCC) (http://www.md-net.org) using the SAS 8 statistical package procedure “PROG PLAN”. After screening for eligibility, patients were randomly assigned at a 1:1 ratio to receive either deflazacort or placebo for the first treatment phase. Patients baseline characteristics with regard to sex, age, body weight and muscle strength assessed by CIDD and HHD did not show relevant differences between treatment groups. The study drug and the placebo preparation were disposed by the hospital pharmacy, a number was communicated to identify the study drug for each patient, and the drug was identically prepacked to maintain the masking for the patient and investigator. To maintain the masking for the trial statistician, reading permission was withdrawn from the computer directory containing randomization information.

The data of all visits were summarized descriptively, using arithmetic mean and standard deviation for numeric data (including changes within each trial period (natural history, crossover periods 1 and 2)) and absolute and relative frequencies for categorical data. For crossover analyses, a mixed model [30,31] was applied with the sequence of the two treatments within the crossover design, the two treatment periods and treatment (deflazacort or placebo) as fixed factors and patients nested within sequence as random factor. This procedure was also chosen to substitute missing values in four patients of this study. The intention-to-treat (ITT) population was used for the efficacy analyses of the two primary endpoints. In addition, a non-parametric procedure25 was applied which allows to assess the treatment effect, the carry-over effect from period 1 to period 2 (utilizing Wilcoxon U-tests) and the period effect (via signed rank tests) within the crossover design. The selection of this analysis procedure was mainly based upon the small sample size of this trial with non-normal distribution of the outcome measures. The per-protocol population (PP) was used for all efficacy analyses of secondary endpoints as specified in the study protocol. This includes all patients who have completed the second crossover period.

#### Sample size considerations

The clinical trial intended to show superior efficacy of deflazacort over placebo in the 2 primary endpoints. The available literature on clinical trials in the investigated indication did not provide sufficiently valid data whereon a statistical sample size calculation could be based. Therefore, sample size calculation was based on effect size consideration. An effect size of d = δ/σ = 0.5 represents a moderate superiority of deflazacort compared to placebo according to Cohen [32]. Effect sizes below d = 0.5 are usually considered as clinically not meaningful, effect sizes of d > 0.8, on the other side, would be associated with clinically relevant benefit of the deflazacort treatment. To demonstrate a difference between deflazacort and placebo with an effect size of at least 0.5 in a crossover design, a number of N = 30 patients were planned to complete the trial with the 2nd crossover period. The following assumptions are made for this sample size determination: α = .0125 (1-sided, adjusted according to Bonferroni-Holm for 2 primary outcome measures), 1-ß = 80%, d = δ/σ ≈ 0.5 and a correlation between the outcome measures in the 2 crossover periods of ρ ≈ 0.6). Allowing a drop-out rate of about 20%, we have aimed for a total sample size of 35 patients. Previous studies in DMD patients using higher daily dose steroid regimen reported drop-out rates of less than 6% [12].

## Results

The recruitment of 35 patients was more difficult than expected; only 25 patients were included between September 2003 and February 2008. However, no patient showed improvement of muscle strength – although blinding was sustained, it became clear, that the study drug did not have a positive effect, and may even have a negative effect on muscle strength. Therefore, a conditional power calculation based on the first 18 patients was performed in February 2008 for answering the following question: is it feasible according to the interim results, that an efficacy of deflazacort versus placebo can be proven, if the clinical trial will be conducted as scheduled (recruitment of 10 more patients)? The result of the power analysis showed that the probability for proving efficacy of deflazacort versus placebo was highly unlikely, even if the clinical trial was continued according to plan. Therefore, we decided to terminate the study and perform a final analysis with all available data (stop for futility). Before the final decision was reached to close the clinical trial, another seven patients were included to the natural course part or proceeded to the medication phase. However, the data of these patients was not included in the interim analysis, since only participants who completed the full trial period were analyzed. In total, 37 patients were screened for this study. Twelve patients could not be included due to loss of ambulation (n = 5), earlier use of steroids (n = 3), lack of compliance (n = 4). Of 25 patients who entered the natural history phase, none discontinued this part of the trial prematurely; two patients had only just finished the natural history phase, when the trial was discontinued; 23 patients were randomized into the crossover treatment. The final analysis population consists of 23 patients, of whom four (patients 12, 13, 21, 23) had not fully completed both treatment phases at the date of the interim analysis, two under deflazacort, and two under placebo.

### Primary efficacy variables

#### Muscle strength assessed by CIDD

**Figure 3:**
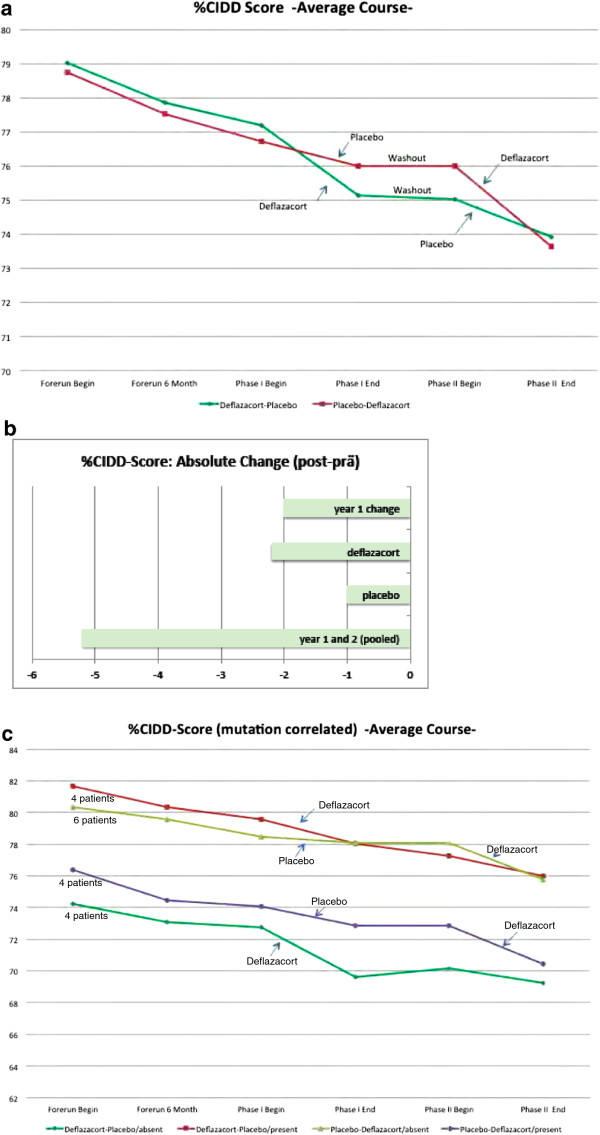
**Manual muscle strength assessed by CIDD in both groups in 18 patients (sequence deflazacort-placebo, and placebo-deflazacort, completer population). a**) Average course%CIDD sum score from natural history until end of both sequences. **b**) Pooled sequences for absolute change%CIDD from natural history until end of both sequences. **c**) Mutation-related CIDD subgroup analysis. Correlation between the type of mutation, resulting in residual dysferlin protein versus total loss of dysferlin protein, assessed by Western Blot and mutation prediction, and outcomes during treatment phases with a sample size of 4 patients in 3 subgroups (sequence deflazacort-placebo, protein present / sequence deflazacort-placebo, protein absent / sequence placebo-deflazacort, protein present) and 6 patients in the remaining subgroup (sequence placebo-deflazacort, protein absent).

Initial testing of manual muscle strength assessed by CIDD in both groups (sequence deflazacort-placebo, and placebo-deflazacort) showed not much difference between the two groups regarding basic muscle strength (between 77% and 80% CIDD). All patients declined during the natural history, at 6 months and even more pronounced at 12 months. In the deflazacort-placebo sequence, patient’s strength declined during deflazacort treatment, partly recovered during the steroid wash-out, but further declined during placebo administration. Patients in the placebo-deflazacort sequence constantly lost strength over the entire trial duration. None of these differences were significant; there was a numerical trend to a slightly larger loss (by 1.2 units in the CIDD) during deflazacort treatment compared to placebo (Figure 3a; change between start and end of pooled crossover periods: deflazacort: d = −2.24; standard deviation (SD) = 3.0; placebo: d = −1.00, SD = 2.2; mixed model result (ITT): p(treatment) = 0.1558; 95% confidence interval [−3.01; 0.52] in disfavor of deflazacort; non-parametric analysis (PP): p = 0.4375). During the one-year natural history, CIDD declined approximately by −2%, during the whole trial duration of 27 months (natural history, both sequences and three-months wash-out), CIDD declined approximately by −5% (Figure 3b).

Assessing percentage of patients with increase or decline of strength as measured by CIDD during natural history and both sequences, we found that during the one-year natural history, 78% of patients declined in strength as measured by CIDD, during six months placebo, 67% of patients deteriorated, but during six months deflazacort, slightly more patients (81%) got worse than during one year of natural course (data not shown).

#### Hand-held dynamometry (HHD)

**Figure 4:**
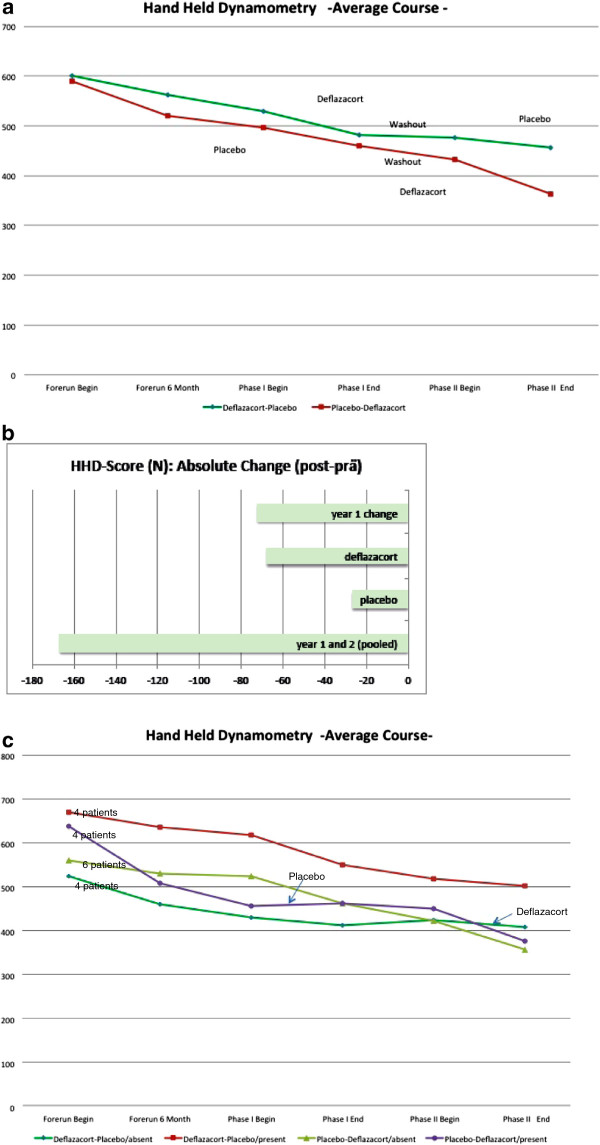
**Hand-held dynamometry (HHD) in both groups in 18 patients (sequence deflazacort-placebo, and placebo-deflazacort, completer population). a**) Average course%HHD sum score from natural history until end of both sequences. **b**) Pooled sequences for absolute change%HHD from natural history until end of both sequences. **c**) Mutation-related HHD subgroup analysis. Correlation between the type of mutation, resulting in residual dysferlin protein versus total loss of dysferlin protein, assessed by Western Blot and mutation prediction, and outcomes during treatment phases with a sample size of 4 patients in 3 subgroups (sequence deflazacort-placebo, protein present / sequence deflazacort-placebo, protein absent / sequence placebo-deflazacort, protein present) and 6 patients in the remaining subgroup (sequence placebo-deflazacort, protein absent).

*HHD* showed that muscle strength in all patients decreased during the natural history, more obvious after 12 than six months. In both treatment sequences, patients strength decreased, partly recovered during the steroid wash-out in sequence deflazacort-placebo, and further decreased during placebo, but more prominent during deflazacort administration (Figure 4a: change between start and end of pooled crossover periods: deflazacort: d = −67.7; SD = 67.6; placebo: d = −26.7, SD = 73.9; mixed model result (ITT): p(treatment) = 0.0546; 95% confidence interval [−76.50; 0.84] in disfavor of deflazacort; non-parametric analysis (PP): p = 0.1309). The pooled sequences for absolute change in HHD showed a decline of 72 N during the one-year observation period and by 167 N during the whole trial duration of 27 months (Figure 4b).

In summary, for none of the primary efficacy variables a statistically significant difference could be demonstrated in this trial. The mean changes and the related 95% confidence intervals indicate a quantitatively larger improvement under placebo compared to Deflazacort.

### Secondary outcome variables

The comparison between deflazacort and placebo in all secondary outcome measures are summarized in Table 1. Changes in each crossover-period were similar under Deflazacort and Placebo. The only two variables showing a slight (p < 0.10) difference between the two treatments are the Vignos Scale and the Hammersmith Motor Ability Score in favor of placebo. In general, the worsening of the scores in all secondary outcome measures which was observed during the 1-year natural course evaluation continued during the 15 months crossover phase under either treatment.

**Table 1:** Comparison of deflazacort and placebo in secondary outcome measures (itt population)

**Outcome measures**	**Baseline**^**†**^	**Natural course**	**Deflazacort (pooled)**	**Placebo (pooled)**	**p-value**
	**(N = 23)**	End of year 1 – begin*	End – begin of crossover*	End – begin of crossover*	**Deflazacort vs. placebo**^**‡**^
		**(N = 23)**	**(N = 21)**	**(N = 21)**	**(N = 19)**
Muscle strength (CIDD)	78.5 ± 9.6 (56–89)	−2.0 ± 2.5	−2.2 ± 3.0	- 1.0 ± 2.2	0.4375
Hand-held dynamometry (HDD) (N)	596 ± 267 (117–1206)	−72.2 ± 109.6	−67.7 ± 67.6	- 26.7 ± 73.9	0.1309
QSMT – Torque Measurement					
Handgrip left (kg)	20.1 ± 10.3 (8–52)	−2.2 ± 3.6	−1.9 ± 2.9	−0.5 ± 2.5	0.4852
Handgrip right (kg)	20.8 ± (10.3) (8–50)	−2.5 ± 3.5	−2.3 ± 2.3	−1.2 ± 2.6	0.7408
Quadriceps (Nm)	136 ± 59 (31–220)	−11.4 ± 90.4	−17.5 ± 55.1	−25.5 ± 49.5	0.9021
Biceps (Nm)	203 ± 133 (11–310)	+12.2 ± 186.9	−36.0 ± 120.6	−63.4 ± 114.4	0.3456
Neuromuscular Symptom Score	35.4 ± 4.9 (23–41)	−0.3 ± 1.6	−2.4 ± 5.0	−0.4 ± 1.5	0.1467
Timed Function Test (sec.)					
Stair climbing	6.8 ± 5,4 (3–25)	+0.8 ± 1.8	+0.3 ± 1.1	+0.5 ± 1.7	0.8478
Running 10 meters	16.9 ± 5.9 (8–30)	+1.6 ± 2.2	+1.8 ± 1.8	+1.0 ± 1.8	0.3689
Getting up from sitting	2.0 ± 2.3 (0.5-10)	+0.3 ± 1.4	+0.4 ± 1.0	+0.6 ± 1.6	0.4875
Getting up from lying	4.1 ± 4,8 (1–20)	+0.5 ± 1.1	+1.3 ± 3.2	+0.5 ± 1.2	0.3909
Vignos Scale	2.4 ± 0.6 (2–4)	+0.2 ± 0.6	+0.3 ± 0.6	0 ± 0.3	0.0712
Hammersmith Motor Ability Score	23.8 ± 6.1 (10–35)	−2.3 ± 3.1	−2.1 ± 2.3	−1.0 ± 1.5	0.0578
Clinical Global Impressions – severity	4.5 ± 1.4 (3–6)	−0.2 ± 1.0	+0.3 ± 0.6	+0.1 ± 1.0	0.3517

†: The column shows arithmetic mean ± standard deviations ± minimum and maximum values.

*: The column shows arithmetic mean ± standard deviations for changes between end and begin of each study period. Deflazacort and placebo values are pooled across both crossover periods.

‡: p-values associated with the treatment effect in Lehmacher’s (1991) proposal for the non-parametric analysis of crossover-data (deflazacort vs. Placebo). Neither carry-over nor period effects were detected in any analysis.

#### Muscle strength measurement assessed by torque measurement (QSMT) and Handgrip force measurement

Handgrip of right and left hand mainly mirrored the results of CIDD and HHD. However, QSMT in biceps and quadriceps muscles bilaterally showed inconsistent changes.

#### Neuromuscular symptom score (NSS)

**Figure 5:**
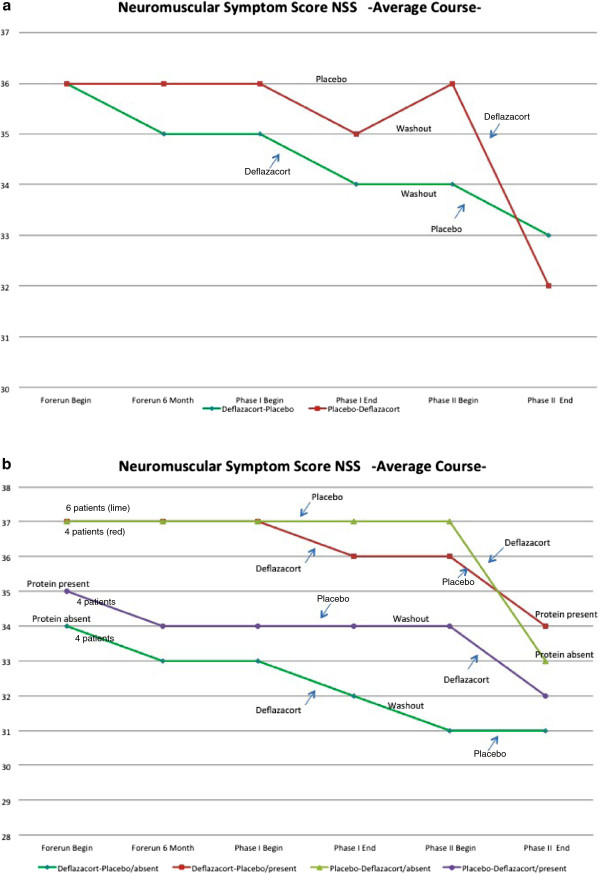
**Neuromuscular Symptom Score (NSS) in both groups in 18 patients (sequence deflazacort-placebo, and placebo-deflazacort, completer population). a**) Average course%NSS sum score from natural history until end of both sequences. **b**) Mutation-related NSS subgroup analysis. Correlation between the type of mutation, resulting in residual dysferlin protein versus total loss of dysferlin protein, assessed by Western Blot and mutation prediction, and outcomes during treatment phases with a sample size of 4 patients in 3 subgroups (sequence deflazacort-placebo, protein present / sequence deflazacort-placebo, protein absent / sequence placebo-deflazacort, protein present) and 6 patients in the remaining subgroup (sequence placebo-deflazacort, protein absent).

The NSS lists 14 daily-life activities and grades them from no disability to severe disability in 4 categories. In both groups, NSS decreased more during deflazacort than during placebo administration, with a trend to recovery during the wash-out in the deflazacort-placebo sequence (Figure 5a).

#### Timed function tests

Timed tests (getting up from lying and sitting position, running 10 m and climbing 4 stairs) showed mild worsening during the complete trial course, but no differences in decline between natural course and treatment phases.

#### Vignos scale and Hammersmith motor ability score

Vignos scale and Hammersmith Motor Ability Score worsened more during deflazacort treatment than under placebo (p = 0.0578 in the non-parametric test for treatment differences). The Hammersmith Motor Ability Score showed overall worsening, not restricted to walking ability.

#### Clinical global impressions (CGI) of change

The global ratings of the disease severity by the investigator did not show relevant differences between the two treatments.

#### Quality of life by SF-36 German version

The subscales and the standardized component scales show the following results taking the range of the scales between 0 (very low quality of life) and 100 (very good quality of life) into account (Table 2):

**Table 2:** Health-related quality of life assessment with the sf-36: baseline data and changes during study phases

**SF-36 scale**	**Baseline**^**†**^	**Natural history**	**Deflazacort (pooled)**	**Placebo (pooled)**	**p-value**
		End of year 1 – begin*	End – begin of crossover*	End – begin of crossover*	**Deflazacort vs. placebo**^**‡**^
Subscales					
Physical functioning	38.3 ± 19.8 (5–85)	−0.1 ± 13.4	−6.8 ± 9.3	+1.5 ± 11.1	0.118
Role Physical	70.6 ± 36.7 (0–100)	+11.8 ± 46.9	−22.4 ± 32.2	+6.6 ± 38.0	0.114
Role Emotional	91.7 ± 22.8 (33–100)	+6.3 ± 25.0	−14.8 ± 46.1	+13.0 ± 34.6	0.037
Social functioning	86.8 ± 12.5 (62–100)	0 ± 12.9	−5.9 ± 23.0	−3.3 ± 14.3	0.963
Mental health	79.8 ± 12.7 (44–96)	−0.2 ± 5.6	−9.1 ± 9.4	+2.9 ± 14.3	0.009
Bodily pain	73.2 ± 16.2 (12–100)	+7.1 ± 21.5	−1.9 ± 25.4	+3.2 ± 18.8	0.394
Vitality	60.0 ± 16.3 (40–95)	+0.3 ± 11.3	−7.4 ± 16.1	+1.8 ± 11.2	0.096
General health perception	45.5 ± 14.4 (25–82)	+6.0 ± 10.9	−4.3 ± 11.6	−1.7 ± 11.0	0.965
Standardized component Scales					
Physical	36.4 ± 8.0 (19–50.4)	+1.5 ± 5.5	−2.5 ± 6.6	+0.4 ± 5.3	0.3123
Mental	56.2 ± 5.8 (40–65)	+0.6 ± 4.6	−4.2 ± 10.7	+1.6 ± 8.7	0.0922

†: The column shows arithmetic mean ± standard deviations (minimum and maximum) values. The scoring of the SF-36 scales ranges from 0 = very bad to 100 = very good quality of life in the different domains of the scale.

*: The column shows arithmetic mean ± standard deviations for changes between end and begin of each study period. Deflazacort and placebo values are pooled across both crossover periods.

‡: p-values associated with the treatment effect in Lehmacher’s (1991) proposal for the non-parametric analysis of crossover-data (deflazacort vs. placebo). With the exception of “role emotional”, there were no carry-over effects detected. No period effects were identified.

At baseline, dysferlinopathy patients reported impairment of physical functioning and in general health perceptions. Compared with a normative sample for the German population25, the physical standardized component scale values were more than one standard deviation below the general population values and are comparable to mean values of patients suffering from other chronic diseases like cardiac diseases (myocardial infarction, angina pectoris), or diabetes. No impairment was seen with regard to the mental standardized component scale and the related SF-36 subscales.

During the 1-year natural history phase, the SF-36 ratings remained largely unchanged or they even improved slightly (physical and emotional role function, bodily pain, and general health perception). Under active treatment with deflazacort, SF-36 ratings in all sub- and component scales worsened as opposed to placebo treatment where the ratings remained stable with a tendency towards a slight improvement in most of the subscales and standardized component scales

Statistical comparison of deflazacort and placebo showed different changes in the subscales mental health (psychological wellbeing) and the role emotional scale (p < 0.05) in favour of placebo. In addition, we observed tendencies towards slightly better quality of life rating under placebo in the vitality subscale and the mental standardized component scale.

#### Mutation-related subgroup analysis

We correlated the type of mutation, resulting in residual dysferlin protein versus total loss of dysferlin protein, assessed by Western Blot and mutation prediction (Figure 1), with outcomes during both treatment phases. We found in both sequences, more pronounced in the placebo-deflazacort sequence, that patients with residual dysferlin protein experienced more worsening than patients with total absence of dysferlin protein (Figure 1, Figure 3c). However, this was not a statistically significant finding, but a trend, which was also seen in the other tests performed.

Worsening of participants with residual dysferlin protein expression under steroid treatment was more evident using the HHD assessment than in the CIDD results (Figure 4c).

NSS worsening is more pronounced in both sequences if residual dysferlin protein is present (Figure 5b). By QSMT, there was no correlation between reduction in strength and residual dysferlin protein (data not shown).

However, the sample size of these subgroups is very small (4 to 6 patients) and therefore these analyses are mainly helpful to generate hypotheses but not a description of possible differences between mutation subgroups.

#### Laboratory findings

CK levels were markedly elevated in all patients, and considerably decreased after deflazacort treatment, but did not correspond with functional ratings (median decrease under deflazacort = −801 units, under placebo = +70 (p = 0.0145). No relevant changes were observed in safety laboratory analytics during the treatment phases.

#### Adverse events

We assessed adverse events in all patients who were treated with study medication (Table 3). We found mild gastrointestinal side effects in four patients (n = 3 on deflazacort, n = 1 on placebo), and weight gain in four patients (n = 3 on deflazacort, n = 1 on placebo). In four patients we saw acne, and two patients suffered from increased blood pressure during deflazacort treatment. In single patients, back pain, Cushingoid features, tremor, fatigue, tachycardia, hair loss, headache and sleep disturbances occurred, all during deflazacort treatment. In six patients, muscle weakness was rapidly progressing during deflazacort treatment, which was not seen in any patient during placebo administration, e.g. patient 4 was no more able to lift his head, while patient 6 could no longer get up from sitting position. Rapid deterioration of muscle strength was noted as an adverse event in all six patients.

**Table 3:** Adverse events during treatment phases

**Patient**	**Adverse events**	**Treatment sequence**	**Treatment phase**
1	Fast progressing muscle weakness, back pain, weight gain (4 kg), acne, Cushing	P/D	Deflazacort
2	None	D/P	
3	Fast progressing muscle weakness, acne, hypertonia, fatigue, tachycardia, tremor	P/D	Deflazacort
4	Fast progressing muscle weakness, mainly in head lifting	D/P	Deflazacort
5	Hypertonia	D/P	Deflazacort
6	Fast progressing muscle weakness, weight gain, Cushing	P/D	Deflazacort
7	Weight gain	D/P	Deflazacort
8	None	D/P	
9	None	P/D	
10	Hair loss, GIT symptoms	P/D	Deflazacort
11	None	P/D	
12	Weight gain (5 kg), acne, GIT symptoms	D/P	Deflazacort
13	GIT symptoms	P/D	Placebo
14	None	P/D	
15	Fast progressing muscle weakness, head ache, sleep disturbances	D/P	Deflazacort
16	None	D/P	
17	None	P/D	
18	GIT symptoms, acne	D/P	Deflazacort
19	Weight gain	P/D	Placebo
20	None	P/D	
21	None	P/D	
22	Fast progressing muscle weakness	D/P	Deflazacort
23	None	D/P	

P = placebo, D = deflazacort, GIT = gastrointestinal.

## Discussion

In conclusion, during one year of natural course, muscle strength declined about 2% as measured by CIDD, and 76 N as measured by HHD. These findings were supported by comparable worsening of the secondary outcome measurements Handgrip, NSS, Vignos Scale and HMAS. Arguably, scores derived from manual muscle testing do not represent interval data; thus one cannot accurately measure percentage change. However, using more quantitative measures of strength demonstrated a similar lack of benefit as did the other secondary outcome measures. Additionally, Vignos and HMAS are fairly rough scales, not suitable for identifying more subtle changes over a one year period in a slowly progressive muscle disease. Vignos Scale and HMAS both showed worsening, slightly in favor of placebo. The Hammersmith Motor Ability Score thereby showed overall worsening, not restricted to walking ability. Interestingly, timed tests did not detect relevant changes illustrating that tests measuring changes in complex activities in milliseconds may not perfectly mirror and have an impact on changes in daily life. Additionally, QSMT by torque measurement die not prove useful to detect reliable changes. Patient-reported quality of life assessments remained fairly unchanged during this first year or improved slightly, mainly in the dimensions, which were impaired in this study population (physical functioning, mental health, standardized physical component scale).

Interestingly, CK levels remained stable during placebo administration, and decreased during deflazacort treatment, but non-corresponding with clinical improvement. This finding confirms that steroids are not just ineffective in dysferlinopathy, but show different activities, thereby enhancing the internal validity of this clinical trial.

Non-recoverable loss of strength in patients with dysferlin-deficiency, who were initially misdiagnosed as inflammatory myopathy and treated with corticosteroids, had been suggested by Hoffmann et al. [17] in an uncontrolled series of 20 patients prior to this study.

Interestingly, we detected reduced dysferlin levels in patients with inflammatory myopathies (polymyositis, inclusion body myositis) treated with steroids (unpublished data). If a reduction in dysferlin-expression would occur in dysferlinopathy patients, then it would affect patients with residual or partially functional dysferlin protein more than patients without any protein. However, we did not obtain muscle biopsies of the patients in our study during or after treatment.

In our trial, patients did not improve during the deflazacort period of treatment. In contrast, under deflazacort treatment, there was a trend to muscle strength worsening, which recovered after discontinuation of the study drug during the wash-out period. In patients with residual dysferlin protein levels, the negative effect of deflazacort was more pronounced than in patients with absent dysferlin protein. However, due to the small sample size of the subgroups, this finding is mainly hypothetic and has not been described before, but we may speculate that steroids have a negative effect on dysferlin expression or function.

In contrast, Belanto et al. [33] hypothesized that a key therapeutic benefit of glucocorticoids may be the up-regulation of dysferlin as an important component of glucocorticoid-enhanced myogenic differentiation. However, this was based on experimentation in cultured C2C12 cells that may not be an adequate model for adult, dystrophic muscle. Further work is required to understand the exact molecular action of steroids on dysferlin expression or function in mammalian muscle, whether it is a direct or an indirect effect.

The SF-36 scores in subscales physical role function and general health perception as well in the standardized physical component scale at baseline showed similar values in dysferlinopathies as in other chronic diseases, and improved slightly during the 1-year natural history phase, possibly due to better patient support and care during the ongoing clinical trial. No impaired quality of life compared to the German general population were observed in the emotional subscales of the SF-36. During deflazacort treatment - in line with the results of the other outcome measures, e.g. strength testing - SF-36 ratings worsened in all subscales, but remained stable or improved slightly during placebo administration, most pronounced for mental health, emotional role and vitality subscales and the standardized mental component scale. The CGI global severity score did not change comparing starting- and endpoint of the trial, suggesting that relevant differences of disease severity were not fully recognizable by the investigator within 27 months of the trial duration.

During deflazacort treatment, we observed a broad spectrum of known side effects. Our study shows that deflazacort is not an effective therapy for dysferlinopathies, and may even harm patients. Therefore, off-label use is not warranted. Even if this result is not what we would have hoped for, it is nevertheless an important finding, since there is good reason now to definitely withhold steroid treatment from patients with dysferlinopathy.

In this study, we were able to establish data regarding the natural course of dysferlinopathies that improve our understanding of the clinical problems these patients are confronted with and may serve as reference for future studies. As measures of muscular function in our sample of juvenile and adult ambulatory patients, CIDD, HHD and NSS have proven useful in showing a change in muscle strength and daily activities within one to two years, while timed tests seemed not suitable. Additionally it became clear that a quality of life measure, specifically adapted on patients with muscle diseases would be warranted, since SF-36 and CGI do not adequately mirror the specific problems of muscular dystrophy patients.

Although no major therapeutic breakthrough has been achieved and curative treatment modalities are not yet applicable, life expectancy and quality of life of dysferlinopathy patients could be remarkably improved by establishing a drug therapy, capable of delaying the dystrophic process and improving muscle strength and function. Unfortunately, no such therapy is available yet, and treatment of dysferlinopathies is mainly based on symptomatic treatment. Therefore, the results of this clinical trial – even being negative - are warranted and may influence further guidelines for steroid treatment in dysferlinopathies. Furthermore, our assessment of the natural history of the disease will provide new insights in the clinical understanding of dysferlinopathies [34].

## Competing interests

This clinical trial was done as a research project of the German network on muscular dystrophies (MD-NET, #01GM0601, research project R19 and S1) funded by the German Ministry of Education and Research (BMBF, Bonn, Germany). MD-NET is a partner of the EU network of excellence TREAT-NMD (EC, 6th FP, proposal #036825). GALENpharma GmbH supported the trial through donation of study drugs. The authors declare that they have no competing interests.

## Authors’ contributions

MCW, MD, MA – study concept and design, supervision, analysis. PR, MD, MA - acquisition of data (patients). ST – acquisition of data (outcome measures). JS, MD – study supervision. HS, MD – acquisition of data (patients). KR, MD – acquisition of data (patients). WK, PhD – acquisition of data (genetics). CMR, PhD – acquisition of data (genetics). MV – acquisition of data (patients). PU, MD – acquisition of data (patients). BS, MD – acquisition of data (patients)’. MD, MD – acquisition of data (patients). BSW, MD - acquisition of data (patients). RK, PhD - statistics. HL, MD – analysis and interpretation, critical revision of the manuscript for important intellectual content. All authors read and approved the final manuscript.

## Authors’ information

*Maggie C. Walter* has trained as a neurologist at the LMU Munich, and is working at the Friedrich-Baur-Institute, the neuromuscular department of the LMU, in leading position as Associate Professor of Neurology. Additionally, she graduated with a master degree in management of social and health institutions. Her main research interests are neuromuscular diseases, mainly muscular dystrophies, myofibrillar myopathies, inflammatory myopathies and clinical trials in neuromuscular patients. Together with Janbernd Kirschner and Matthias Vorgerd, she is coordinator of the German Muscular Dystrophy Network (MD-NET), funded by the Federal Ministry of Education and Research (BMBF) since 2003, member of the TREAT-NMD governing board and TACT from the very beginning, and member of the new TREAT-NMD alliance. She is responsible for the German patient registries, the DMD, SMA and soon-to-come CMT registry, and together with Volker Straub for the international FKRP registry. Since 1997, she is member of the Scientific Advisory Board of the Muscular Dystrophy Association of Germany (DGM), and ad hoc reviewer for Telethon and several peer-reviewed journals.

*Hanns Lochmüller* is Professor Hanns Lochmüller trained as a neurologist in Munich (Germany) and Montreal (Canada). He was appointed chair of experimental myology in the neuromuscular research group at the Institute of Genetic Medicine of Newcastle University in 2007. Hanns has a longstanding interest in the molecular genetics of the inherited myopathies and neuromuscular junction disorders, and is interested in the further study of animal models of these disorders as a means to understand their pathophysiology as well as to develop means to monitor disease progression and therapeutic interventions. Ongoing work in these areas in cell and animal models of muscular dystrophy is concentrating on gene transfer, pharmacological interventions and cell therapy. Hanns is co-founder and former coordinator of the German muscular dystrophy network (MD-NET), and scientific coordinator of EuroBioBank, a European network of biobanks for rare disorders. He led the activity on “patient registries and biobanks” for TREAT-NMD answas elected Chair of the TREAT-NMD Alliance Executive Committee in April 2012.

## Statistical analysis

Gabriele Ihorst, IMBI Institute for Medical Biometry and Informatics, Freiburg, Germany and Ralf Kohnen, RPS Research Germany GmbH, Nürnberg, Germany.
